# Botulinum Toxin Intervention in Cerebral Palsy-Induced Spasticity Management: Projected and Contradictory Effects on Skeletal Muscles

**DOI:** 10.3390/toxins14110772

**Published:** 2022-11-08

**Authors:** Cemre Su Kaya Keles, Filiz Ates

**Affiliations:** Experimental Biomechanics Group, Institute of Structural Mechanics and Dynamics in Aerospace Engineering, University of Stuttgart, Pfaffenwaldring 27, 70569 Stuttgart, Germany

**Keywords:** botulinum toxin, cerebral palsy, spastic muscle, spasticity management, active muscle, passive muscle, muscle structure, muscle morphology, muscle biochemistry

## Abstract

Spasticity, following the neurological disorder of cerebral palsy (CP), describes a pathological condition, the central feature of which is involuntary and prolonged muscle contraction. The persistent resistance of spastic muscles to stretching is often followed by structural and mechanical changes in musculature. This leads to functional limitations at the respective joint. Focal injection of botulinum toxin type-A (BTX-A) is effectively used to manage spasticity and improve the quality of life of the patients. By blocking acetylcholine release at the neuromuscular junction and causing temporary muscle paralysis, BTX-A aims to reduce spasticity and hereby improve joint function. However, recent studies have indicated some contradictory effects such as increased muscle stiffness or a narrower range of active force production. The potential of these toxin- and atrophy-related alterations in worsening the condition of spastic muscles that are already subjected to changes should be further investigated and quantified. By focusing on the effects of BTX-A on muscle biomechanics and overall function in children with CP, this review deals with which of these goals have been achieved and to what extent, and what can await us in the future.

## 1. Introduction

The most common motor disability in childhood, cerebral palsy (CP), describes a condition in which damage to the part of the brain that controls body coordination ultimately impairs the functioning of the musculoskeletal system. Spasticity affects most patients with CP, and the focal injection of botulinum toxin type-A (BTX-A) into the affected muscles is one of the techniques used in the management of spasticity. By causing muscle paralysis, BTX-A aims to diminish spasticity and thereby improve joint function but has been shown to lead to some unexpected effects.

The most obvious effects of BTX-A injection on a muscular level are shown as weakness and atrophy [1,2]. Importantly, excessive weakness as one of the most widely reported adverse effects [3,4] causes an independent risk factor for joint degeneration which further can lead to osteoarthritis [5,6]. Most importantly, because children with spastic CP (SCP) already have muscular defects (i.e., weakness), a treatment that potentially leads to further weakening is already a concern [7]. Some studies [8,9] have suggested that impaired muscle growth rather than spastic overactivity may influence the development of contractures, and BTX-A exposure may do more harm than good. Therefore, not only the functional effects of BTX-A observed in clinical practice, but its biomechanical effects on muscular structures, need to be considered in the time course of therapy.

This study presents a general understanding of CP-induced spasticity and the techniques used in its management, a review of the projected and contradictory effects of BTX-A administration on muscle function and structure, and brings together the latest findings to shed light on its future use.

## 2. Cerebral Palsy-Induced Spasticity and Techniques for Its Management

Skeletal muscle is the motor that generates motion and maintains posture by exerting forces. A muscle has tension even at rest due to the presence of several contracting motor units [10]. When a muscle stretches and the active contractions reach enough tension to produce movement, the neuromuscular system can respond by changing muscle tone [11]. There are contractile and viscoelastic components (i.e., active and passive components, respectively) of muscle tone, which are mathematically expressed as the changes in resistance or force per tissue displacement and biologically maintained by the complex interplay of spinal and supraspinal mechanisms [12]. The passive components of muscle tone are independent of neural activity, but depend on factors such as connective tissue and sarcomeric non-contractile proteins such as titin; thus, the active component results from motor unit activations [12,13,14]. Therefore, the key to the muscle action of contraction is the regulation of stretch reflexes over which the central nervous system has control. With the activation of stretch reflexes, the generation of muscle tone is performed by muscle spindles based on the contraction of intra- and extrafusal fibers according to commands from the brain to the spinal cord [15]. Spasticity, the involuntary and repetitive contraction of skeletal muscles, may arise following upper motor neuron syndrome. It represents a condition in which stretch reflexes are exaggerated due to reduced inhibition in a more noticeable way with faster movements [16]. This results in the co-occurrence of undesirable muscle activation which is thought to be due to a loss of control of motor neurons and can further contribute to muscle hypertonia [10,11,17]. Spasticity-related hypertonia leads to prolonged muscle contraction, i.e., persistently increased resistance to stretch [18], which reduces the muscles’ ability to lengthen and impairs their growth [19,20].

A sustained shortened state of the spastic muscle may cause contracture [21,22] and develop bony deformities [23], which can further result in elevated joint and muscle stiffness [21,24,25,26,27], and a limited passive and active joint range of motion (ROM) [28,29,30]. Therefore, the abnormality in motor control in spasticity disrupts the voluntary activation of skeletal muscle, leading to a musculoskeletal pathology and resulting in functional limitations.

Spasticity can stem from many etiologies [17], including CP, which describes a group of permanent motor disorders attributed to damage to the central control system in the developing fetal or infant brain [31,32]. Although brain lesions causing neurological conditions are not progressive, the secondary muscular pathology resulting from abnormal motor control persists and progresses through the lifespan [33]. Today, CP is the most prevalent of all childhood motor disabilities, occurring in up to 3 and 3.6 cases per 1000 live births across Europe [34] and the US [35], respectively. As one feature of spasticity [33,36], most children with CP develop hypertonia. The spastic type of CP accounts for 77–86% of all cases [35,37] and the spastic muscles of patients differ from those of typically developing (TD) people morphologically, biomechanically, and thus, functionally.

### 2.1. Spastic Muscle

**Muscle volume** reduction in the paretic [29,38,39,40,41,42,43,44,45,46] and even in non-paretic limbs [29,40] has consistently been reported in individuals with SCP relative to TD individuals. The deficits in muscle volume as an indicator of overall muscle growth [47] are observed in the spastic muscles of children with CP as early as the age of 15 months [48].

**Muscle thickness and the cross-sectional area** also decreased as other size abnormalities due to SCP [29,33,46,47,49,50,51,52]. The physiological cross-sectional area (PCSA) represents the sum of cross-sectional areas of all the muscle fibers and can be estimated as the ratio of muscle volume to fascicle length and corrected by the pennation angle [53]. Accordingly, fascicle length and angle are additional morphological parameters to be considered in PCSA calculations. However, the comparisons of fascicle length and angle between the paretic and non-paretic muscles have produced inconsistent findings [51,54,55]. Given that the differences in muscle volume tend to be more pronounced than the differences observed in muscle fascicle lengths [40], the major determinant of reduced PCSA, and thus, of force production capacity of muscle, has been suggested as the reduced muscle volume [47].

**Muscle–tendon unit length** is also impaired with SCP, in addition to the aforementioned parameters. Several studies have shown that the spastic gastrocnemius has a shorter muscle belly compared with that of the non-paretic side [40] or in TD people [39,56]. A shorter muscle is one of the indicators of contracture formation [57]. However, the force-generating capacity of a muscle, among other things, is dependent on not only the muscular features but also the morphology and architecture of the corresponding tendon [58]. The examination of length changes in the muscle-tendon unit of the spastic gastrocnemius revealed a longer-than-normal Achilles tendon for the patients [59,60]. It has been shown that children with CP, even without muscle contracture and who are not restricted in their performance of daily tasks, exhibit morphological alterations of the plantar flexor muscle-tendon unit [61], which has been suggested as a contributor to functional deficits such as muscle weakness.

**Muscle weakness**, an impairment of the ability to generate or maintain a required level of force [62], is a common clinical symptom in children with SCP [29,63] and is considered the major interfering factor in gait [64]. The decreased muscle size leads the muscle strength to change at the joint of children with SCP in comparison with TD children. However, the contribution of deficits in muscle size to muscle weakness is not constant; rather, it shows notable heterogeneity between joint movements and between subjects [65]. Therefore, it mainly has a neural basis. Studies have questioned the relationship between lower-limb muscle weakness versus reported pathological gait parameters, for instance, that the weakness in the dorsiflexors and plantar flexors in children with CP compared with those in TD children was associated with delayed and decreased knee flexion angle during the swing, respectively [66]. For unilateral and bilateral SCP groups, in particular, significant correlations were found between the combined sagittal motion of the pelvis, hip, knee, and ankle joints and the composite weakness scores of all involved muscles, for the majority and the entire range of the gait cycle, respectively [67].

Apart from neural basis, the changing intrinsic capacity of a muscle also modifies its strength [29,63]. Elder et al. [29] explained the weakness in ankle plantar flexors in children with spastic hemiplegia with a smaller cross-sectional area and inability of full activation of the muscles, as well as the co-activation of antagonists. Later findings in the quadriceps femoris and triceps surae [68] supported that muscle weakness is accompanied by decreased agonist voluntary muscle activation as well as greater antagonist co-activation.

**Antagonist co-activation** has been known to be more pronounced in patients with CP for over 20 years [69,70] and is considered necessary for joint stability because patients have major motor coordination problems. However, it can also cause hampered movement due to increased joint stiffness [69,70]. Recent intraoperative experiments [71,72,73,74] demonstrated that co-activation of the antagonistic muscles can adversely affect the force characteristics of the spastic muscle to agree with the pathological knee joint condition in patients with SCP.

**Muscle and connective tissue stiffness** were also shown to be increased due to SCP. For example, Vaz et al. [75] found that poor performance in the hand function of children with hemiplegic SCP was correlated with wrist muscle weakness and flexor stiffness. For flexor carpi ulnaris muscles, de Bruin et al. [25] showed a sizable thickening of the tertiary perimysium, which suggests elevated stiffness of the connective tissues. Using imaging techniques [76,77,78] or performing muscle biopsy analysis and quantifying changes in intramuscular connective tissues [27,79], many other studies have also revealed increased stiffness for various muscles such as the gastrocnemius, gracilis, semitendinosus, and vastus lateralis in patients with SCP compared with those in healthy subjects. Therefore, increased muscle stiffness reflects the changes in both mechanical and biological properties of the muscles, meaning that all the intrinsic muscular properties (e.g., myofiber stiffness) [80], and the amount [27] and orientation [25] of connective tissue structures affect the muscular stiffness rather than it being only and completely related to the muscle size alterations (i.e., deficits in muscle cross-sectional area or length) observed in SCP.

### 2.2. Spasticity Management

In CP, there is no cure available for brain injury, leading to motor problems characterizing the disease. Thus, motor disability persists throughout the lifespan and alters its presentation with time due to normal development and the aging process [81]. All the interventions represent management techniques, each of which aims to mitigate a particular impairment, with the assumption that functional improvements will succeed. The main determinant in the selection of management techniques in spasticity is determined by focusing on the symptom and its underlying factor. Due to muscle weakness, many patients attempt to maintain posture and movement using exaggerated muscle tone. By reducing that tone and allowing the target muscle to stretch, the development of muscle contractures is aimed to be minimized. The expectation is to eventually widen the joint range of motion and facilitate independent movement in one’s daily life. The technique(s) to be used need to be compared in terms of risks (e.g., limited benefit, muscle atrophy, muscle weakness, and adverse effects) and benefits (e.g., decreased spasticity, improved ROM, and decreased stiffness) and optimized using an integrated approach for highly efficient and successful treatment in each individual patient [19,82]. Although the distribution and severity of symptoms vary widely among individuals with CP, current treatment approaches for motor disabilities mainly consist of non-pharmacological, medical, and surgical therapies and are used to improve joint ROM and therefore motor function, by normalizing muscle tone, inhibiting abnormal reflexes, and/or spastic agonist muscles, and strengthening weak muscles.

**Non-pharmacological therapy modalities** are available for ambulatory children with SCP. Commonly used physical therapy interventions can be exemplified as neurodevelopmental therapy and conventional physical therapy. The usage of adjunct therapies such as electrical stimulation [83] and kinesio-taping [84], in combination with physical therapies, attracts attention to enhance postural [85,86] and sitting [87] control in patients with SCP. Various techniques like muscle stretching [88], muscle strength training [89,90], training of exercises [91] and gait [92], serial castings [93], and the use of orthoses and assistive devices [94], keeping in mind that the administration of any of these alone might generally not be very effective in reducing muscle tone or stiffness or improving gait parameters [95,96,97,98,99].

**Selective dorsal rhizotomy** (SDR) is a surgical intervention to reduce lower-limb spasticity [100], which has been reported to provide an increased ROM accompanied by moderate improvements in body structure and function, and in daily living activities, even after 20 years of the operation [101,102,103,104]. In contrast to the clinically significant effects [105], some groups presented results suggesting that the long-term benefits are questionable. For instance, the work of Tedroff et al. [106] prospectively examining children with SCP for 10 years after SDR reported that there was a slight recurrence of spasticity at the knee and ankle joints, and joint ROM and median ambulatory status were best 3 years after SDR and then declined; therefore, they suggested that SDR alone does not prevent contracture development in the long term, or, in other words, that contracture development is not mediated solely by spasticity.

**Orthopedic surgeries** are considered in SCP if there is a significant fixed contracture at the joint that causes rotational problems if the patient has a curvature of the spine that causes problems with sitting up or walking, or if there are pain or hygiene problems secondary to any of the above [81]. Neurectomy, tenotomy, osteotomy, muscle lengthening, muscle and tendon transfers, tendon release, and arthrodesis are major types of orthopedic surgeries performed in children with CP [81,107]. However, surgical operations are irreversible, the recovery could take long and it can be painful, and the results are variable [108]; thus, delaying the need for surgery is important in the management of spasticity.

**Pharmacological options** come to the fore for this very purpose. Among the numerous oral medications such as tizanidine, diazepam, clonidine, and dantrolene, the most commonly used for hypertonia is Baclofen [107,109,110]. However, all are with different mechanisms of action and side effects, especially sedation, which is not well tolerated in children [110,111]. Intrathecal administration of Baclofen rather than oral administration has been shown to reduce spasticity in children with CP with far lower doses required orally and fewer side effects [110,112,113]. However, due to the short half-life of the drug, it has to be infused continuously, and the identification criteria for deciding whether the patient is suitable for such therapy are not clear completely [107].

Intramuscular injections are applied to produce neuromuscular blockade. The injections of neurolytic agents, alcohol (45–100%), and/or phenol (3–7%) have been reported to reduce spasticity for a longer duration [107,114]. However, as there is a risk of permanent muscle fibrosis and painful dysesthesias, the location of the motor point must be exactly determined [110,115,116].

When focal spasticity is considered, the most commonly and effectively used treatment is the intramuscular injection of the botulinum toxin. Compared with phenol injections, toxin therapy provides better functional improvements [117] and is associated with fewer complications because the effects are selective and reversible [118]. In the 1990s, the use of botulinum toxin in the treatment of spasticity observed in children with CP was introduced. Koman et al. [119] presented successful toxin injection as a potentially valuable tool that can delay surgical intervention until the child is older and at less risk of possible complications, including the need for repetitive surgeries. Since then, the clinical use of the toxin in CP has grown in a very short time and rapidly [20], as have the publications and dissemination of consensus statements [120,121,122,123]. There is a rationale that botulinum toxin injection, with its effect of reduction in spasticity, opens a “therapeutic window” for treatment in general, which may support, for example, facilitating muscle stretching therapy or fitting orthoses and casts [124]. The toxin administration, therefore, aims to prevent contracture formation, enhance motor ability, improve posture and functional skills such as self-care, and provide better pain management [82,125,126].

Pain, as a health condition, affects most patients with CP and inter-relates with their mental health and quality of life (e.g., less attention and participation in daily living, and sleep problems) [127,128,129]. Dickinson et al. [130] explained that self-reported pain primarily determines the patients’ decreased life quality, which causes up to 7% variation in the management of their condition. Any intervention in patients with SCP should be driven by specific aims, which can be broadly grouped as improved posture and function, facilitation of care, and management of pain [82], and what the patient’s primary needs are for treatment is a highly variable subject that requires careful decision-making. The effect of botulinum toxin in releasing spasticity-associated pain is, therefore, worth considering. For instance, preoperative toxin injection into the surgical muscle was shown to reduce postoperative pain after hip adductor-release surgery in patients with SCP [131]. This effect was reported to be so big that it reduced the need for analgesics by 50% and the mean pain score by 74%. A recent study [132] assessed patients with SCP who experience at least modest pain during passive range of motion before and after a single lower extremity intramuscular toxin injection with frequent intervals. The intervention was reported to cause a significant and clinically meaningful decrease in localized and daily pain for 28 weeks. This mediated improvements in function as well, such that the functional goals evaluated by the goal attainment scale (SMART GAS [132,133]) were significantly achieved, yielding an obvious short-term effect on the quality of life. The analgesic efficacy of botulinum toxin in children with SCP, however, varies across gross motor function classification system (GMFCS) levels, and rigorous methodological quality is required for a definitive inference [127,134]. Leaving aside such activity and participation parameters, which are usually clinically followed by questionnaires and clinical scales, but of course, have great importance on the health status of patients, this review focuses on the quantitative changes in structural and functional properties that occur after focal botulinum toxin injection.

When considering botulinum toxin as a treatment option, patient selection and timing of the treatment are critical. The primary criterion for identifying suitable patients for toxin injection is the presence of either persistent or dynamic hypertonia, in the absence of significant fixed deformity [135]. The focal indications of the botulinum toxin injection have been well recognized and described [120], such as a dynamic equinus or a dynamic knee flexion angle persistent throughout the gait cycle in the lower limb, or a persistent thumb in the palm or thumb adduction in the upper limb [135]. However, in either of them, it should be noted and properly assessed that relative muscle weakness may be a contraindication for toxin therapy [135]. Although most botulinum toxin-treated children will eventually require corrective surgery, delaying the need for surgery until 6–12 years of age has a clear advantage, allowing surgical interventions to be fewer and more precise [136]. Therefore, as a maximum outcome and prolonged effects which can only be taken before the development of any fixed and severe contractures, early injections in younger children are preferable. Early therapy reduces and may prevent the formation of contractures and thus delay surgery, whereas this is not the case in older children who show limited and short-lived responses due to the presence of fixed contractures [137,138,139]. The optimal timing for treatment, therefore, is recommended as 2 to 5 years of age, a range agreed upon by most scientists, when dynamic motor development continues, and gait patterns are flexible so there is still a chance to modify the course of the disease [136,137,140,141]. It should also be noted that the variability in the amount of response to BTX-A treatment varies between and within the patients. Defining goal attainment scaling to evaluate the treatment, Desloovere et al. [142] searched for delineating features of treatment success and failure, which resulted in 7.1% of the patients having an unsuccessful response to the treatment, meaning that they had unmet some specific goals based on gait or upper limb assessment, clinical examination, and other parameters such as hygiene and pain when compared pre- and post-evaluations. The lack of response may have been caused both by the age of the patient (older patients with low responsiveness) and the level of the injection (children with low responsiveness receive more BTX-A injections in the proximal parts of the lower limb and fewer multilevel injections compared with children with high responsiveness) or the therapy (i.e., casting) applied in combination with BTX-A treatment [142]. By focusing on the effects of botulinum toxin on muscle biomechanics and overall function in children with CP, this review deals with which of these goals have been achieved and to what extent, and what can await us in the future.

## 3. Mechanism of Botulinum Toxin Administration

The botulinum toxins are protein exotoxin products of the bacterium *Clostridium botulinum* [143]. Of the seven major serotypes of botulinum toxin (A to G) with varying properties (e.g., muscle-weakening efficacy, duration of action, and the protein targeted), botulinum toxin type-A (BTX-A) has been introduced as optimum for long-term clinical use [144,145]. The activation mechanism of BTX-A is based on its effect on the process named exocytosis which allows the release of acetylcholine into the synaptic cleft at the neuromuscular junction and requires the involvement of several specific proteins. This process ultimately causes muscle fibers to contract because of the change in the electrical potential of the membrane it causes [146]. Therefore, any intervention preventing the discharge of acetylcholine-containing vesicles at the neuromuscular junction, namely, preventing exocytosis, results in presynaptic blocking in signal transmission, and thus, causes muscle paralysis [146,147,148,149,150,151]. Without acetylcholine release, muscle fibers cannot be physiologically activated [3]. This is what happens with focal BTX-A injections and is used as such to help counteract the pathologically altered spinal excitatory and inhibitory circuitry leading to increased excitation and decreased inhibition observed in spasticity.

Over the course of time, the formation of new axonal sprouts takes place and vesicle recycling occurs in these sprouts but not at the parent nerve terminals. Then, the cholinergic function of the original terminals is restored in the long run, and the paralysis eventually disappears [152]. Thus, the action of BTX-A (i.e., muscle paralysis) is reversible. The degree of muscle paralysis is, therefore, dependent on the number of synapses affected which is also based on the applied dose and volume of the toxin [7,120,153]. The re-establishment of functional parent nerve terminals is evident to begin 4 weeks after BTX-A injection [152]. After about 3 months, the sprouting of new nerve endings involved in neurotransmission’s initial recovery disappears as the original nerve endings regain their ability to release acetylcholine [152]. In clinical practice, the relaxation effect of BTX-A lasts from 12 to 16 weeks in most patients [135]. An accurately applied injection procedure is vital for obtaining elevated treatment outcomes for longer periods and decreased side effects such as the spread of the toxin.

Chemo-denervation of proprioception: The spasticity-decreasing effect of BTX-A is based on induced muscle weakness which occurs due to the extrafusal effects of the toxin. However, Phadke et al. [154] showed that BTX-A induces significant chemo-denervation of intrafusal muscle fibers in addition to extrafusal fibers, which indicates that when the intrafusal effects of BTX-A therapy are maximized and extrafusal effects are minimized, the therapy would be more effective in reducing spasticity while decreasing the side effect of muscle weakness in the treatment area as well. Moreover, the clinical effect of BTX-A appears to continue beyond inducing muscular weakness [155]. The reduction in hypertonia can be because of synaptic blocking occurring due to the interaction of BTX-A with both intrafusal and extrafusal fibers of the injected muscle, as well as the inhibition of the stretch reflex loop [156,157]. Not only the spinal cord but also the central nervous system can be affected by toxin injections. Examining the cortical responses and the stretch reflex responses (with the evaluation of cortical somatosensory-evoked potential (SEP) and soleus H wave, respectively) in children with SCP, Frascarelli et al. [155] demonstrated neurophysiological changes induced by BTX-A and confirmed the key role played by rapid conductive afferent fibers, especially proprioceptive fibers, in the therapeutic management of motion and lower-limb functions. Based on the findings, they concluded that spasticity appears as a factor affecting the cortical SEPs, and even though BTX-A did not seem to have any direct effect on sensory pathways, the authors supposed an indirect mechanism on modulation of specific afferent fibers due to the modification induced by BTX-A to central loop reflex.

## 4. Effects of BTX-A Administration

### 4.1. Active Muscle Properties in Relation to Movement

The main goal of BTX-A treatment is to reduce the exaggerated muscle tone and hyperactive stretch reflexes seen in patients with CP, thus improving joint function. This is mainly accomplished by reducing the active force production of muscles exposed to BTX-A [158,159,160,161].

Table 1 exemplifies the effects of BTX-A administration on the related joint function assessed in patients with CP.

**Acute and short-term changes:** Previous studies have shown that many of the kinematic and kinetic parameters measured in patients with CP approximated healthy-person gait in about 1.5 months after BTX-A injection into the calf muscles [138,163,166,168]. BTX-A injected into the gastrocnemius improves the ankle and knee movements 5 days post-injection [163]. It is important to note that not only are the distal joints directly associated with the injected muscle but the proximal joints might also be affected. For example, after injection into the gastrocnemius, acute worsening of hip joint motion has been reported [163]. The initial excessive hip flexion observed was to compensate for the improved knee position and obtain better stability. Hip power and pelvic tilt also worsened. Consistent with these findings, in another study, BTX-A injected into the gastrocnemius increased the ROM of the knee during the gait cycle, but the ROM of the hip worsened, and the hip joint power increased significantly [166]. It was noted that the knee joint probably began to do so to amplify the reduced push-off ability after the BTX-A injection [166]. However, a general improvement in the overall gait pattern has been demonstrated after the second injection for about one year, as the hip and knee joint ROMs increased during gait, and hip power in the stance phase reduced due to the decreased hip flexion [163]. By changing the joint mechanics of the lower-limb muscles, BTX-A further alters the trunk movement, such that greater trunk excursions were found in the transverse plane 4 months after BTX-A treatment [164]. That time is clinically relevant because the reduced spasticity due to BTX-A is accompanied by improved ankle angle at initial contact, maximal and mean ankle dorsiflexion in the stance and the swing phases, along with a significant reduction in maximal ankle plantarflexion at push-off [162]. Dynamic foot pressure measurement demonstrated that the foot contact pattern (e.g., foot contact area, and the contact length and width of the hindfoot) and plantar pressure distributions are also improved, suggesting better heel contact. However, regarding foot progression, no significant changes in the mean and maximal internal rotation during the entire gait cycle were found.

By investigating gait function over time after BTX-A treatment, Matsuda et al. [168] concluded that the maximum improvement in gait function does not occur during the early stage, instead approximately 2 months after the treatment. A recent study [169] showed significant improvements in ankle gait kinematics after BTX-A injection into the gastrocnemius and/or semitendinosus, without a significant improvement in knee kinematics in the sagittal plane. However, the gait profile score was found to be decreased following the treatment which implies improvements in overall gait pathology. In their longitudinal study, Löwing et al. [167] revealed that the improvement in gait provided by BTX-A injection applied with goal-directed physiotherapy was significant but clinically small. The clinical assessment indicated that plantar flexor spasticity was reduced after 3 months and remained stable, whereas passive ankle dorsiflexion increased after 3 months but decreased slightly after 12 months. Goal attainment gradually increased, reached the highest levels at 12 months, and levels were maintained at 24 months. The identified spasticity reduction effect of BTX-A, however, did not have a positive effect on improvements in gait or goal attainment. Therefore, the clinical significance of the improved gait remained unclear. In another longitudinal study, Eek and Himmelman [165] demonstrated that 6 months after injection, a small improvement in knee extension at initial contact was obtained, but no changes in ankle angle in the gait analysis were found. Regarding voluntary muscle strength, it has been found that voluntary force production in plantar flexor muscles did not decrease after BTX-A at 6 weeks after injection; instead, there was a trend of increased muscle strength at follow-up. Consistent with other studies [173], it can be explained that the blocking of involuntary nerve impulses leads to an opportunity to use and train the muscles with voluntary control [165]. Notably, the gait-related findings were different from previously reported findings of improved peak ankle dorsiflexion in stance after BTX-A [171]. Gait pattern improved regarding knee extension at initial contact in the legs with BTX-A treatment in the gastrocnemius, but there were no changes in ankle angle in the gait analysis.

On the other hand, the available literature shows that no improvement in the spatiotemporal parameters (i.e., step length, stride length, cadence, gait velocity) occurs after BTX-A, regardless of the time elapsed after treatment, in general [138,165,166,171]. However, apart from its effects on muscle spasticity and its possible functional repercussions, BTX-A treatment is a good option to test surgical prospects. The muscle-weakening effect of BTX-A injection can be used to determine whether the patient’s function is dependent on the strength of a particular target muscle, and thus, to plan its surgical lengthening by avoiding poor outcomes [170]. Careful selection of patients is essential for surgical operations because remarkable side effects have been reported to occur [174]. Using pre-operative BTX-A injections, Rutz et al. [170] showed that 20.9% of patients were at risk of functional deterioration (i.e., excessive ankle dorsiflexion at the terminal stance of the gait cycle, loss of knee extension at the mid and terminal stance, and/or increased anterior pelvic tilt during the whole gait cycle) in cases of muscle weakening. This proportion is in line with the results of a historical cohort study [170] in which previous surgical outcomes were analyzed and showed deterioration in 18% of patients after hamstring- and Achilles-tendon-lengthening surgeries. Therefore, BTX-A injections can be used as a reliable tool for filtering out patients who are at risk of deterioration after lengthening surgery.

**Long-term effects:** The gait pattern in the sagittal plane of children who receive multilevel BTX-A treatment for an average of 1 year 10 months after the initial injection shows more normal anterior pelvic tilt, along with improved hip and knee kinematics at the end of stance and more normalized ankle motion [138]. These indicate that the gait pattern is less defined by muscle contractures at the level of the hip, knee, and ankle. In the transverse plane, the gait deviations (i.e., internal hip rotation at initial contact, toe-off, and mid-swing) were more pronounced in the control group who did not receive BTX-A treatment. This suggests that children treated with BTX-A have a gait pattern that is less defined by bony deformities, common in SCP. Therefore, based on these results, BTX-A injection can be suggested as a technique to decrease the possibility of secondary problems at early ages and minimize the need for complex surgery at a later age. However, other long-term follow-up studies have reported conflicting results [138,172,175]. Tedroff et al. [175] indicated that the contractures continue to develop in the long term despite the muscle tone reductions induced by BTX-A. In a later study [172], two groups of children with CP received a daily stretching program, and children in the BTX-A group additionally received two injections, 6 months apart in the gastrocnemius muscle. After a 1-year treatment phase of the study, the subsequent treatment of each child was pragmatically and individually designed (including additional BTX-A injections into the muscles of children in both groups). It was found that plantar flexor muscle tone in the BTX-A group was significantly reduced after 3.5 years, whereas the muscle tone at the ankle and knee in the control group remained unchanged. The knee joint ROM was significantly increased at 1 year in the BTX-A group but reduced at the knee and ankle joints in the control group after 3.5 years. No group differences were found for gait analysis, GMFM-66, or PEDI. The authors concluded that early BTX-A treatments during 1 year in toddlers with CP reduce muscle tone and arrest the progression of contractures compared with the control group. However, the long-term functional effect is less clear. Notably, the effects of the serial lower limb intramuscular BTX-A injections on the gait quality of children with bilateral SCP, who were naïve to BTX-A at baseline, were assessed using the Edinburgh Visual Gait Score; the findings showed that the improvements following the first injection, with their gait quality, were maintained following the second and third injection cycles [176]. Similarly, in a different longitudinal study [177] testing the efficiency of multiply BTX-A therapy in patients with SCP for up to eight injection sessions, the treatment gain was found to be the highest up to 3 months after injection, and the number of injections did not impact the effectiveness. Thus, the effectiveness of repeated BTX-A injections, which are expected to result in functional improvements by affecting muscle mechanics, should be carefully reconsidered. Therefore, the diagnosis and follow-up of muscle level parameters, where functional changes in patients may have been fundamental, is very necessary.

### 4.2. Passive Muscle Properties

Toxin injection affects the passive stretch reflexes and results in altered active as well as passive muscle biomechanical properties. The influence of BTX-A on the passive properties of the injected muscles has been assessed in various studies (Table 2).

**Muscle stiffness:** Some in vivo studies reported a decrease in mouse muscle stiffness 1 week after chemical denervation [184,185,186]; however, Pingel et al. [187] showed that it did not change acutely. In contrast, it tended to increase in the 2-month post-injection period. The elevated in vitro stiffness for rat muscle fiber bundles 1-month post-injection was also reported [188]. Apart from the relative increase in stiffness of elastic elements, a tendency for an increase in reflex-mediated stiffness following BTX-A injection has also been reported (but without reaching statistical significance) [187]. This was explained by possible compensatory neuroplastic adaptation. It should be noted that stiffness increase is not in accordance with the purpose of using BTX-A as an efficient anti-spastic medication that releases muscle tension and helps prevent contracture development.

**Figure 1:**
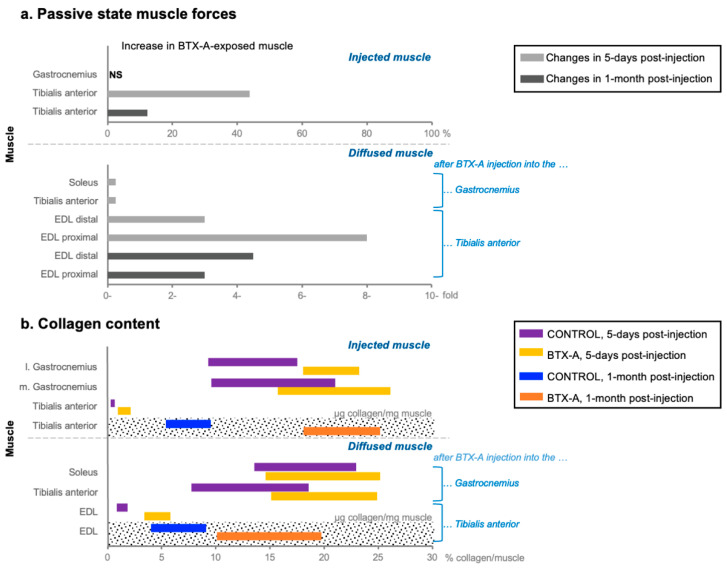
Passive muscle properties of rat hindlimb muscles are illustrated based on [159,160,161,190,192,193]. (**a**) Increases in passive (i.e., resting) state muscle forces in BTX-A exposed muscles compared with those of the control group The bars indicate that passive state forces increase in different percentages at different muscle lengths, and maximal increments are presented. EDL: extensor digitorum longus; NS indicates changes are non-significant. (**b**) Intramuscular collagen amounts of both BTX-A exposed and control group muscles. Data for both the injected and non-injected but diffused muscles due to the spread of the toxin are illustrated. The dotted shaded graph areas represent the studies in which the amount of collagen was quantitatively measured, whereas, in other areas, the percentages of ECM presented relative to fiber content.

**Figure 2:**
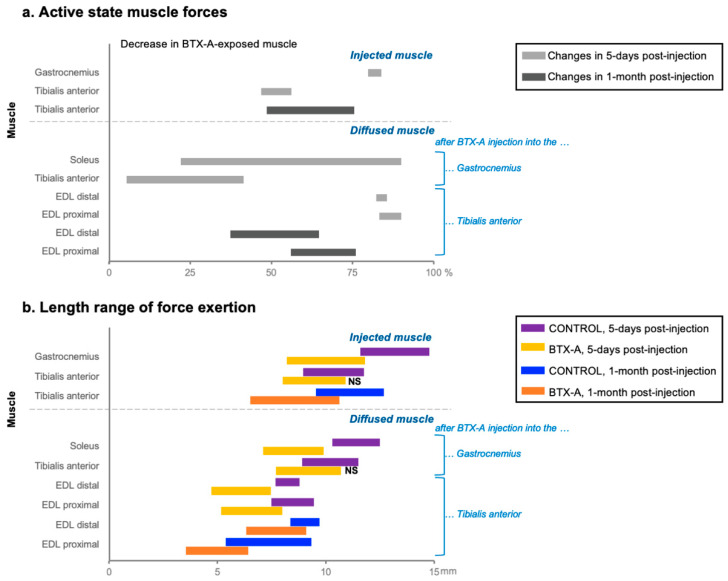
Active muscle properties of rat hindlimb muscles are illustrated based on [159,160,161,190,192,193]. (**a**) Decreases in active state muscle forces in BTX-A exposed muscles compared with those of the control group. The bars indicate that active state forces decrease in different percentages at different muscle lengths. EDL: extensor digitorum longus. (**b**) The muscle length range of force exertion of both BTX-A exposed and control group muscles. Data for both the injected and non-injected but diffused muscles due to the spread of the toxin are illustrated. NS indicates that changes are non-significant.

**Passive muscle force:** BTX-A administration affects the passive force amplitudes (muscle resistance at the resting state), depending on the time elapsed after injection and the length of the muscle [186,189]. Experiments in rat anterior crural compartments have shown that BTX-A elevates the passive forces of the injected tibialis anterior muscles acutely [161], and 1 month after injection [159] (the time period which allows testing the long-term effects of BTX-A in animals, by avoiding exocytosis in the parent terminals [152] for consistent testing [3,158,188]). Five days after injection, passive force increase has been observed only at longer muscle lengths, whereas 1 month after injection, the passive forces were higher for the BTX-A group compared with those of the control, for all lengths tested [159,161]. Remarkably, those changes have been observed with a decrease in mass and an increase in intramuscular collagen (i.e., the main load bearing constitute of the extracellular matrix, ECM) of the injected muscles (Figure 1) [159,190], and in concert with the elevated ECM stiffness considered in previous finite element modeling studies, investigating the time course of BTX-A treatment [191]. In these studies, no widening of the injected tibialis anterior muscle’s length range of active force exertion was reported (Figure 2), suggesting that the changes in the muscle’s mechanics may not be a direct improvement in functionality [161]. In contrast to the clinical expectation, BTX-A has been reported to cause even a narrower length range of force production, at 1-month post-injection [159]. All these changes demonstrated in animal studies make it vital to better understand the effects of BTX-A administration on human muscle mechanics and their relation to joint function.

**Studies on human muscles’ passive resistance to stretch:** BTX-A treatment in CP was reported to demonstrate great success in reducing the passive motion resistance between 3 and 16 weeks after injection, followed by an improvement in motor status [182]. However, a longer time was needed for movement skills to be regained. Using an integrated assessment based on EMG and torque, Bar-On et al. [179] revealed the BTX-A-induced improvements for nearly all EMG and torque parameters measured during manually performed passive stretches of the medial hamstrings at high velocity and at high versus low velocity; however, large inter-subject variability was noted.

Alhusaini et al. [178] investigated how the intrinsic properties of spastic muscles change during the stretch-shortening after BTX-A. They reported that the toxin did not lead to any decrease in passive intrinsic muscle stiffness and hysteresis. The findings were similar to that of a previously reported study [28], indicating that compared with TD children, children with CP exhibit around threefold higher passive stiffness in the calf muscles. Although BTX-A reduced the stretch hyperactivity (i.e., neurally mediated responses), the unaltered intrinsic stiffness of the muscle continued to offer significant resistance to passive movement of the ankle. The case study performed on children with CP [183] using a dynamic sonoelastography indicated decreased muscle stiffness together with a decrease in spasticity and an improved GMFM score, 4 weeks after intensive rehabilitation technique with BTX-A injection to the gastrocnemius. The authors attributed different results from the previous finding [178] to the intensive rehabilitation technique applied after BTX-A injection.

A relatively new and non-invasive approach to analyzing the stiffness of tissues is ultrasound shear wave elastography. The muscle elastic modulus was shown to represent local passive forces [194] and be linearly related to active muscle force [195]; therefore, this approach is suitable for examining adaptations of skeletal muscles as a result of a disease or an applied treatment. Using shear wave measurements, greater passive muscle stiffness of lateral [77] and medial gastrocnemius muscles [196], as well as biceps brachii as an elbow flexor muscle [78], was shown for children with CP. In a pilot study [181], quantifying the effects of BTX-A on passive muscle properties in children with CP, a temporary, but statistically significant decrease in passive lateral gastrocnemius muscle stiffness at some specific ankle joint positions between 1 and 3 months post-BTX-A was found, although with no significant improvement in ankle passive ROM or spasticity. This, therefore, suggests that BTX-A-induced changes in passive muscle stiffness may not lead to clinically meaningful improvements in patient mobility. A longitudinal study [180] showed a similar result, that BTX-A causes a decrease in stiffness of gastrocnemius only in the first month after injection. The effect disappears in a time period of 3 months.

### 4.3. Muscle Structure, Morphology, and Biochemistry

Despite the clinical outcomes due to BTX-A treatment having been reported, studies on the structural changes of BTX-A-exposed muscles are relatively scarce. Particularly, pioneering animal studies have highlighted the need to accurately detect the detrimental effects of BTX-A at the level of the spastic muscle.

**Atrophy:** Just as spastic paretic muscles are subject to changes in their structure, BTX-A application leads to further alterations. The biggest concern is muscle atrophy and problems with overall muscle growth. Many animal studies have shown that the weights of BTX-A-treated muscles are significantly reduced within the acute period [159,190,197]. It is known that BTX-A puts forward the expectation of spasticity reduction as a secondary effect of such muscle atrophy [1], during which the contractile elements of the muscle are partially replaced by fat and connective tissue [198]. However, there are studies showing that even after the paralysis effect of BTX-A has disappeared, morphological recovery is still incomplete. Remarkably, Ma et al. [199] showed that rat gastrocnemius muscle did not attain normal size within the 12-month observation period, after a single BTX-A injection. The muscle atrophy caused by repeated administration of BTX-A injections in rabbits showed that the initial exposure resulted in the greatest reduction [200], with such changes (i.e., altered muscle mass and/or contractile material) in both the injected and also non-target muscles (in contralateral limbs) could not fully recover even after 6 months of repeated monthly BTX-A injections [201].

**Impaired muscle growth:** BTX-A-induced structural adaptations in muscles have been demonstrated in human muscles as well [198]. Impaired muscle growth (based on, e.g., muscle volume and muscle thickness) has been reported to occur within 1 to 3 months after injection [202,203]. Importantly, after this time interval, although the pharmacological paralysis effect of BTX-A on the muscles is expected to disappear [135,152], morphological changes in the muscle continue to be observed in the next period. In longitudinal follow-up studies analyzing the muscle atrophy after BTX-A treatment in children with CP, muscle volume reductions have been reported for injected psoas [204] and medial gastrocnemius [205] muscles, even after 6 months of injection. A recent study examined the morphology of the medial gastrocnemius muscles in children with CP, again 6 months post-injection, and by comparing the muscle growth of BTX-A-naïve children with that of children who remained BTX-A-naïve after 6 months [206]. The authors reported hampered muscle growth which was primarily attributed to the reductions in muscle cross-sectional dimensions, whereas growth in the muscle length was preserved over time. However, a 1-year prospective investigation of changes in the medial gastrocnemius muscle morphology in children with SCP who were also naïve to previous BTX-A treatment showed that both the cross-sectional and longitudinal muscle growth (assessed in terms of muscle volume, fascicle length, and PCSA) continues to occur, and regardless of the injection frequency (single vs. multiple injections) [207]. However, at the end of the 1-year post-injection period, medial gastrocnemius muscle volume and PCSA in children with SCP receiving BTX-A injections remained significantly lower than in TD children (both for the single and multiple injection groups), which has been noted as a potential contributor to the muscle weakness and thus needs to be considered in terms of the long-term effects on muscle growth. Such findings suggesting BTX-A-induced hampered muscle growth are quite remarkable because the alterations in skeletal muscle growth are already a concern in patients with SCP, which points out the risk of a further worsening.

**Changes in biochemical properties:** The denervation subsequent to BTX-A-induced paralysis results in the expression of a neural cell adhesion molecule (N-CAM: a membrane glycoprotein that accumulates on muscle fibers after denervation and is not expressed in untreated muscle) [208]. The level of N-CAM on injected mouse tibialis anterior muscle was found to be increased at 2 weeks post-injection and remained detectable even after 1 month, whereas by 2 months it had dropped to barely detectable levels [208]. Importantly, N-CAM expression in non-injected muscles soleus, gastrocnemius, and quadriceps followed the same trend as that of tibialis anterior, indicating the diffusion of BTX-A. When muscles are denervated, it is generally reported that this causes a shift in the myosin heavy chain (MHC: the distribution of its isoforms is a key determinant of fiber shortening velocity used in the categorization of fiber type and has a major influence on maximal force production) composition toward faster, type II fibers and fewer slow, type I fibers (i.e., decreased percentage of slow, type I muscle fibers and increased percentage of fast, types IIa, IIx, and IIb muscle fibers) in both slow and fast muscles [209,210]. The study by Dodd et al. [189] on rat triceps surae muscles injected with BTX-A, in contrast, showed findings that do not support that. Ten weeks after injection, type I MHC increased both in injected and in the contralateral muscles, with additional differences in other types II fibers of MHC as well, which shows that BTX-A paralysis causes muscle fiber atrophy in both the injected as well as contralateral muscles and strikingly indicates similarities to those seen with aging, not to those seen with denervation.

**Molecular Level Changes:** Analysis of the gene expression of BTX-A-injected rat tibialis anterior muscles over 1 year showed important changes, with dramatic transcriptional changes in the first week (compared with 4, 12, and 52 weeks after injection) [211]. The genes regulated at 1 week cover a wide spectrum of functions, such as stabilizing the neuromuscular junction, sarcomeric contraction, and muscle metabolism (e.g., maintenance of calcium homeostasis). Of the whole genes regulated exclusively at 4 weeks, most were associated with ECM and collagen fibril organization, which showed general upregulation. Confirming this, higher collagen levels have been reported in muscle biopsy studies for such a time range after injection [159,188]. Collagen as well as titin are remarkable in understanding and relating structural changes to muscle atrophy. The protein, titin, is important for the structural and mechanical stability of the sarcomeres, and the reduction in its content induces lower sarcomere stability and muscle atrophy [212]. For instance, 3 weeks after injection, BTX-A in rat gastrocnemius muscles reduced the titin content by 18% [213]. Such changes occurring at the muscle level are significant, and it is critical for a muscle to recover from fibrosis and atrophy caused by denervation from BTX-A exposure and to balance these muscular changes with the clinical benefits of the treatment.

## 5. Recent Findings on the Effects of BTX-A

**Length dependency of force reduction:** Active force drop due to BTX-A has been shown in various animal studies [159,160,161]. These findings were also notable, as BTX-A led the muscle force to reduce not equally for all lengths studied, but rather differentially (Figure 2a). Previously, Longino et al. [3] showed that BTX-A-induced muscle weakness in rabbit knee extensors is greater in short compared with long muscle lengths. Studies on rat hindlimb muscles [159,160,161,190,193] showing higher force reduction at low muscle lengths were in accordance. Such length-dependency affects the related joint torque production and suggests that the effects of the toxin on joint mechanics are complex and just as hard to control. New studies combining isometric and dynamic in vivo muscle force measurements are needed to investigate whether the length-dependent effects could explain some of the discrepancies shown in gait due to BTX-A.

**Partial paralysis:** The functional weakness targeted by BTX-A injection is a result of its localized paralyzing effect. Thus, in cases of complete paralysis, it would not be possible for a muscle to contract and generate movement at all. After partial paralysis with a toxin, the response to tetanic contraction remains normal in shape but diminished in proportion to the decrease in twitch tension [214]. In rat tibialis anterior muscle, Shaari and Sanders [215] quantified muscle paralysis by electrically stimulating the fibular nerve that innervates the tibialis anterior and then stained muscle sections for glycogen (fiber areas containing glycogen represented the regions of BTX-A action). The most effective paralysis was reported to occur when the injections were given directly into the motor endplate region (i.e., the mid-belly and the closest to the superior protuberance of the tibia), whereas injections farther from this point resulted in a decrease in paralysis. The area of glycogen-stained fibers decreases as the distance from the mid-belly increases, and remarkably, the injection was given inferior to the mid-belly causing a large-speckled region of paralysis, most probably representing partial paralysis. After BTX-A injection into the rat tibialis anterior, partial glycogen-staining was shown not only for the injected but also for other compartmental muscles due to toxin diffusion (see Figure 3 published in Ates et al. [190]), indicating partial paralysis in all exposed muscles.

**“Longer sarcomere” Effect:** Recent animal studies [159,160,161,190,192] reporting complex and adverse effects of the BTX-A treatment (e.g., muscle-length-dependent force reduction, narrowing of the muscle’s length range of force exertion rather than widening, etc.) suggest the need for further research on partial paralysis [216]. Using the finite element approach, the partial paralyzing effect of BTX-A was modeled (by not activating half of the fascicles from different locations of the modeled muscle) and analyzed versus a BTX-A-free muscle model (see Figure 1, Figure 2 and Figure 3 published in Turkoglu et al. [216]). The study indicated that sarcomeres within muscles exposed to BTX-A attained longer lengths (i.e., do not shorten) compared with the identical sarcomeres of a BTX-A-free muscle [216]. Due to the muscle fiber–ECM interactions present, this effect is also reflected in the activated fibers [217], and activated muscle parts show an enhanced potential of active force exertion. Therefore, due to the longer sarcomere effect, a muscle force reduction originating exclusively from the paralyzed muscle fibers is compromised by the changes in active sarcomeres leading to a smaller net force reduction which varies as a function of muscle length as well [216]. This leads the sarcomeres to reach their maximal force production earlier, causing the muscle’s optimum length to shift to a shorter length [216], thus explaining the narrowing in BTX-A-exposed muscle’s length range of force exertion found in animal studies [159,160,190]. The altered conditions in sarcomeres within active parts of the partially paralyzed muscle are, therefore, responsible for complex mechanical effects to occur. Furthermore, the model prediction of such effects of BTX-A may become more pronounced in the long term due to increased stiffness of the muscle’s ECM [191]. This has recently been proven with a long-term study on rat tibialis anterior muscle having a narrower range of force exertion [159].

**Enhanced collagen amount and myofascial force transmission:** The elevated muscular collagen indicating the increased stiffness of the ECM structures is one quite remarkable adverse effect of BTX-A treatment, which most probably alters the force transmission mechanism as well. In addition to the myotendinous force transmission, myofascial force transmission could be altered remarkably. These two different types of force transmission describe the direct and inverse pathways, respectively, and are simply distinguished by whether the force is transmitted through the tendons or fascia. Among the different types, epimuscular myofascial force transmission (EMFT), in particular, is defined as the transmission of force generated within a muscle to the skeleton by pathways other than the muscular origin and insertion (i.e., bypassing the myotendinous pathway) via connective tissue linkages [218,219,220]. EMFT through such a fascial network of connective tissue structures between activated muscles has been shown to occur and change the force amplitude and/or the range of force exertion of a muscle in animals [221,222,223,224], healthy individuals [225,226,227], and as well as patients with SCP [71,72,73,74,228]. Studies [229,230,231] in which in vivo human muscle forces were directly measured showed that selectively activated spastic knee flexors produce only low forces when flexed, and high forces in the extended knee joint positions (thus, the force curves for spastic muscles show qualitative similarities with the forces of healthy human muscle) [232], which could not reveal the source of exaggerated flexor forces forcefully keeping the joint at flexed joint positions, and causing the pathology of restricted joint motion. However, recent studies analyzing the muscle force–joint angle relationship in children with CP reported that EMFT significantly increases the force of spastic knee flexors [71,72,228] (e.g., about 33% for a primary knee flexor muscle, semitendinosus) and is in line with patients’ pathological gait patterns [73,74]. EMFT in CP, therefore, has clinical implications, but its physiological significance is a matter of debate because its effects have not yet been directly quantified in comparison with healthy subjects.

Moreover, recent studies reported that BTX-A has significant effects on EMFT. One characteristic effect of EMFT is the proximo-distal force difference which describes the unequal forces exerted at both ends of a biarticular muscle [233]. The alterations in the directions and/or amplitudes of the myofascial loads (e.g., because of the stretching of the intermuscular connections due to muscle relative position changes) make a difference in the target muscle’s net force amount, which is what happens with local BTX-A injections. Previously, Yucesoy et al. [234] showed that BTX-A injected into the mid-belly of the rat tibialis anterior affects the proximo-distal force differences for the neighboring extensor digitorum longus muscle in the acute term. A specific test involving solely relative position changes in the extensor digitorum longus, although it was being kept at a constant length, resulted in minimized proximo-distal force differences for extensor digitorum longus, thus indicating diminished EMFT after BTX-A exposure [234]. Conversely, a similar study, performed this time a long time after injection, showed that such EMFT was unchanged compared with the control group [193]. Notably, decreased active and increased passive forces were reported for the injected muscle in both these post-injection periods compared with those in the control group, although it was shown that the length range of motion of the target muscle was only narrowed in the long term after injection [159,161]. This indicated that acute adverse effects of BTX-A persist and advance in the long term. Combining these mechanical alterations with diminished and unaffected EMFT findings of diffused muscles 5 days and 1 month [193,234] post-BTX-A injection, respectively, one can suggest that the acute efficacy of BTX-A is likely based on the suppression of EMFT, which finally returns to its pre-injected scenario. Most importantly, how and for how long these effects would present in humans are unknown. Recently, utilizing ultrasound shear wave elastography, Ates et al. [227] quantified localized changes in the shear modulus [195] of lower-limb muscles during ankle movements performed at two different knee angles and demonstrated the existence of EMFT via intermuscular mechanical interactions for healthy adults. The effects of BTX-A application on the stiffness of connective tissue structures and EMFT through them should be considered for the better management of spasticity. Accordingly, for investigating whether intermuscular mechanical interactions and thus EMFT, differ in patients with CP compared with healthy individuals and in patients before and after BTX-A exposure, applying ultrasound shear wave elastography in clinical practice can potentially be a useful tool, for both understanding CP and monitoring the muscular changes during the time-course of therapeutic interventions, all of which will undoubtedly lead to findings that will have great clinical repercussions.

## 6. Conclusions and Future of BTX-A Use in CP

In patients with SCP, the main goal of BTX-A treatment is to improve joint function by reducing spasticity-associated muscle hypertonia. There are numerous studies showing that properly administered BTX-A therapy leads to effective improvements with limited side effects. However, management of the alterations in the muscles’ mechanical characteristics (e.g., strength and stiffness) due to BTX-A exposure during the time course of the treatment is critical. The contradictory effects that deteriorate the condition of the muscles that are already subjected to changes due to SCP (Table 3) should be carefully considered. However, many of the only studies focus on specific parameters (i.e., obtained by investigating active and/or passive muscle characteristics, or focusing solely on morphology rather than function), and therefore, conclusive evidence is lacking to directly link the holistic effects of the treatment at the muscle level and the mechanical action of the involved joints. Conflicting findings in the literature point to this problem. Overall improvement in the patients’ gait pattern is one major challenging goal in clinical practice, which requires the consideration of many features simultaneously because BTX-A results in variations in kinetic and kinematic parameters in joints not only at the distal but also at the proximal side of the injected muscle.

For suitable patient groups, BTX-A reduces the development of permanent contractures and related structural deteriorations; therefore, it could postpone the need for surgical interventions. However, the recent findings on animal muscles conflict with clinical expectations. A consistently shown contraindicated effect is that BTX-A does not widen the muscles’ length range of force exertion and does not reduce the muscles’ passive stiffness, even though it has great efficiency in decreasing the muscle tone in dynamic conditions. While continuing to benefit from the projected effects of the BTX-A intervention, it is vital to somehow minimize or even eliminate its adverse effects, which possibly stem from structural alterations such as the elevated amount of collagen in BTX-A exposed muscles in comparison with those of controls. Further studies are needed to determine how adverse effects on muscle mechanics and connective tissue structures due to BTX-A administration, which has been previously revealed by animal studies, change directly in humans selectively, and also when certain combined treatments are applied simultaneously. Investigating such BTX-A-induced alterations will enable the more accurate planning of adjuvant therapies (e.g., exercise, electrical stimulation, orthoses, serial casting, taping, etc.) [235,236,237,238] used in the management of spasticity in CP. This urges more detailed human studies that would simultaneously examine the muscular changes with joint function, and ultimately provide the possible pharmacological development of BTX-A therapy based on the newly obtained findings.

## Figures and Tables

**Table 1:** Selected studies representative of the changes in the movement of patients with CP after BTX-A treatment.

Reference	Study Population	Aim/Intervention	Muscle(s) Injected	Duration of Observation	Outcome Measures	Results
Choi et al. [162]	25 children with hemiplegic (*n* = 17) and diplegic (*n* = 8) SCP. GMFCS: I or II	To investigate the effect of BTX-A injection on gait and dynamic foot pressure distribution in children with SCP	Gastrocnemius and TP	Before and 1 and 4 months after injection	-Clinical assessment using the MAS and MTS -3D gait -Dynamic foot pressure measurements with F-scan system	Spasticity reduced, dynamic foot pressure and ankle movement improved at both 1 and 4 months after BTX-A.
Cimolin et al. [163]	A child with spastic diplegic CP (4-year-old)	To quantify the effects of BTX-A in reducing excessive knee flexion due to calf spasticity/Two injections at an interval of 10 months	Gastrocnemius	Before injection, 5 days after the first injection (POST1) and 3 months after the second (POST2) BTX-A	-3D gait	POST1: Ankle and knee dynamic ROM improved, but hip position and hip power worsened. POST2: All joints tested improved
Degelaen et al. [164]	28 children with spastic hemiplegic (*n* = 14) and diplegic (*n* = 14) CP (3–12 years) GMFCS: I or II	To analyze the effect of BTX-A on trunk postural control and lower-limb coordination	Lower limb muscles (psoas, hip adductors, medial hamstrings, gastrocnemius, soleus)	Before and 4 months after injection	-3D gait	Lower-limb coordination is unaltered. Varying effects (decrease/increase) in trunk movement. Greater trunk excursions in the transverse plane.
Desloovere et al. [138]	60 children with SCP (BTX-A = 30, control = 30, 19 with hemiplegia, and 11 with diplegia in each group) GMFCS: I-III	To evaluate the effects of multilevel BTX-A treatments on the gait pattern of children with SCP/Both groups received the same conservative treatment, in the BTX-A group children received min one BTX-A.	Lower limb muscles (gastrocnemius, hamstrings, adductors, iliopsoas, TP, less often, RF, soleus, tensor fascia latae, foot muscles)	An average of 1 year 10 months after the initial injection	-3D gait combined with EMG	The BTX-A group showed a less pronounced pathological gait pattern. Major improvements were pelvic anterior tilt, max hip and knee extension, and internal hip rotation.
Eek and Himmelmann [165]	20 children with spastic hemiplegic (*n* = 16) and diplegic (*n* = 4) CP GMFCS: I or II	To detect if voluntary muscle strength is affected by BTX-A injections, and relate the effect of BTX-A to gait pattern and passive ROM	Gastrocnemius	Before, and 1.5 and 6 months after injection	-Clinical assessment using the MAS -Muscle strength measurement with a handheld device -2D gait	A small increase in passive ROM. Increased muscle strength after 6 months. Improved knee extension at initial contact. No changes in dynamic ankle angle, gait velocity, or stride length.
Galli et al. [166]	15 children with hemiplegic (*n* = 8) and diplegic (*n* = 7) CP (5–11 years) 20 TD children (5–14 years)	To evaluate the BTX-A-induced improvement of the walking functional ability in children with CP	Gastrocnemius	Before and 1.5 months after injection	-3D gait	Improvement in the ankle, knee, and hip joint positions and ROMs at gait. The peak absorbed ankle power reached values close to those of the control group.
Löwing et al. [167]	40 children with spastic hemiplegic (*n* = 24) and diplegic (*n* = 16) CP (4–12 years) GMFCS: I or II	To evaluate short and long-term effects of BTX-A combined with goal-directed physiotherapy in children with CP/1 to 10 injections were given	Mainly plantar flexors	Before and at 3, 12, and 24 months after the initial injection	-3D gait -GAS and body function assessment	A significant but small long-term improvement in gait. GAS increased, reached the highest levels at 12 months, maintained at 24 months.
Matsuda et al. [168]	9 children with hemiplegic (*n* = 1) and diplegic (*n* = 8) CP (4–8 years) GMFCS: I, III, and IV	To investigate the gait function over time after BTX-A treatment	Gastrocnemius Additionally, soleus, hip adductors, RF, hamstrings, TP, and upper limb muscles	Before and 1, 2, and 3 months after injection	-Clinical assessment using the MTS and ROM -2D gait using the Foot Contact Scale and the Physician’s Rating Scale -GMFM-66	Significant improvements for the max dorsiflexion at gait, the ankle ROM, and GMFM-66 in 8 weeks. Increased knee joint extension torque in 12 weeks.
Peeters et al. [169]	45 children with SCP (BTX-A = 25, control = 20, 20 with hemiplegia, and 25 with diplegia) (3–11 years) GMFCS: I-III	To investigate the impact of BTX-A on muscle morphology, spasticity, and gait	Gastrocnemius and semitendinosus	Before and 8–10 weeks after injection	-3D gait (in BTX-A group) -EMG during passive muscle stretches (in BTX-A group) -3D ultrasound (BTX-A vs. control)	Significant improvements in ankle kinematics. Limited effects on knee kinematics. Spasticity reduced. Muscle volumes reduced only in BTX-A group.
Rutz et al. [170]	110 children with CP. 42 hemiplegic, 39 diplegic, 27 quadriplegic patients and 2 with ataxia. GMFCS: I-III	Application of BTX-A before a muscle lengthening surgery to test whether muscular weakness deteriorates function hence the gait pattern	All muscles considered for surgical lengthening	1.5 and 4 months after injection	-3D gait analysis combined with EMG	20.9% (*n* = 23) showed deterioration in gait after pre-operative BTX-A test injections resulting in the cancelation of their lengthening surgeries.
Sutherland et al. [171]	20 children with CP (2–16 years) 10 with hemiplegia, 9 with diplegia, and 1 with quadriplegia.	To quantify the gait of subjects receiving two injections of either BTX-A (*n* = 10) or saline (*n* = 10)/Two injections were given at an interval of 1 month	Gastrocnemius	Before and 2 months after the first injection	-3D gait analysis, gait video recordings, and dynamic EMG	Dynamic ankle ROM improved in the BTX-A group. No difference in time-distance parameters, and dynamic EMG. Muscle strength change varied between subjects.
Tedroff et al. [172]	15 children with hemiplegic (*n* = 6) and diplegic (*n* = 9) CP. GMFCS: I-III	To evaluate the subjects receiving a daily stretching program with (*n* = 6) and without (*n* = 9) 1-year of repeated BTX-A treatment/Two injections given at an interval of 6 months.	Gastrocnemius	Before and 1 and an average of 3.5 years after the initial injection.	-Clinical assessment using the MAS -3D gait -GMFM-66 -PEDI	Reduced plantar flexor muscle tone in the BTX-A group. No group differences at gait, GMFM-66, or PEDI.

Gray-shaded references contain multiple injections of BTX-A. EMG: electromyogram; GAS: goal-attainment scaling; GMFCS: Gross Motor Function Classification System; GMFM-66: Gross Motor Function Measure; SCP: spastic cerebral palsy; MAS: Modified Ashworth scale; MTS: Modified Tardieu scale; PEDI: The Pediatric Evaluation of Disability Inventory; ROM: range of motion; RF: rectus femoris; TP: tibialis posterior.

**Table 2:** Characteristics of included studies that represent the changes in the passive muscle properties due to BTX-A treatment.

Reference	Study Population	Aim/Intervention	Muscle(s) Injected	Duration of Observation	Outcome Measures	Results
Alhusaini et al. [178]	16 children with spastic CP (4–10 years) GMFCS: I or II	To investigate non-neurally mediated calf-muscle tightness before and after BTX-A injection	Gastrocnemius and soleus	Before and 6 weeks after injection	-Passive ROM -Joint torques at predetermined angles -Passive mechanical stiffness, and hysteresis	An increase in ankle passive ROM, a small decrease in the torque required to achieve plantigrade and 5° of dorsiflexion. No change in ankle torque. No difference in myotendinous stiffness or hysteresis
Bar-On et al. [179]	19 children with hemiplegic (*n* = 6), diplegic (*n* = 11) and quadriplegic (*n* = 2) CP (3–18 years) GMFCS: I-IV	To quantify the effects of BTX-A injection in treating medial hamstrings spasticity	Medial hamstrings	Before and 43 ± 16 days after injection	-Position, torque, and EMG signals integrated during passive stretches of the medial hamstrings at low, medium, and high velocity.	Improvements found for nearly all EMG and torque parameters at high velocity and at high versus low velocity, however large inter-subject variability was noted.
Bertan et al. [180]	33 children with CP received either a BTX-A injection and a home-based exercise program (*n* = 17) or only a home-based exercise program (*n* = 16).	To investigate the stiffness of the gastrocnemius with sonoelastography after BTX-A injection and to examine the relationship with clinical parameters	Gastrocnemius	Before and 1 and 3 months after injection	-Ankle passive ROM, the MAS, and MTS evaluation -Pediatric Functional Independence Measure, and GMFCS -Muscle stiffness assessed by ultrasound elastography	Muscle stiffness decreased in the BTX-A group only in the first month after treatment. MAS, MTS, and passive ROM improved but not GMFCS. A correlation between the clinical and elastographic measurements.
Brandenburg et al. [181]	10 children with spastic CP (2–12 years) GMFCS: I-III	To quantify the effect of BTX-A on passive muscle properties using ultrasound shear wave elastography	Gastrocnemius	Before and 1 and 3 months after injection	-Ankle passive ROM, and the MAS evaluation -GMFCS -Muscle stiffness assessed by B-mode ultrasound and shear wave elastography	No differences in ankle passive ROM or spasticity. Passive muscle stiffness dropped at some specific ankle joint positions between 1 and 3 months post-BTX-A.
Colovic et al. [182]	27 children with spastic CP (2–6 years) GMFCS: I-III	To evaluate the effects of BTX-A on passive motion resistance of the affected muscles and the functional motor status	Lower limb muscles: Group I (*n* = 16) injected into their adductors, hamstrings, gastrocnemius. Group II (*n* = 11) injected into the gastrocnemius	Before and 3, 8, 16 weeks, and 6 months after injection	-Passive motion resistance estimated using the MAS -Achieved functional motor level evaluated by GMFCS and GMFM	Decreased passive motion resistance for hip adductors and knee extensors in Group I over 3–16 weeks, and for ankle joint extensors in both groups. Both groups: GMFCS improved 16 weeks after injection, GMFM increased after 8 and 16 weeks.
Kwon et al. [183]	A child with spastic diplegic CP (28-month-old)	To show the change in muscle intrinsic stiffness due to BTX-A treatment/Intensive rehabilitation performed	Gastrocnemius	Before and 4 weeks after injection	-Passive motion resistance using the MAS -GMFM -Muscle stiffness by B-mode ultrasound and dynamic sonoelastography	Improved MAS and increased GMFM score. Decreased muscle stiffness shown by a decrease in shear velocity, and an increase in the strain ratio.

EMG: electromyography; GMFCS: Gross Motor Function Classification System; GMFM: Gross Motor Function Measure; MAS: Modified Ashworth scale; MTS: Modified Tardieu scale; ROM: range of motion.

**Table 3:** The contradictory effects of BTX-A intervention on skeletal muscles.

Spastic Muscles in Comparison with Healthy Muscles	BTX-A Exposed Muscles in Comparison with Control Muscles
**Impaired muscle growth**	
Muscle size alterations	Muscle atrophy
smaller volume [29,38,39,40,41,42,43,44,45,46]	reduced volume [203,204,205]
less thickness [33,47,49,50]	reduced thickness [202]
smaller cross-sectional area [29,33,46,47,49,50,52]	reduced muscle mass [159,190,197,199,200]
Muscle-tendon unit length alterations	Reduced length range [159,160]
shorter muscle belly [39,40,56]	
longer tendon [59,60]	
**Structural adaptations**	
Greater collagen [25,27,79]	Increased collagen [159,160,190]
Greater stiffness [75,76,77,78,196]	Increased passive state forces [159,161]
**Functional deficits**	
Muscle weakness (less strength) [29,63,67,75]	Muscle weakness (reduced force production in active state) [159,160,161]
Disrupted co-working of muscles	Altered mechanics of diffused muscles [190,192,193]
less agonist activation [68]	
greater antagonist activation [29,68,69,70]	

## Data Availability

The data presented in the figures through a pooling of previous publications are available from the corresponding author upon reasonable request.
